# Annotations

**Published:** 1892-09-03

**Authors:** 


### ANNOTATIONS.

**Jewish and Gentile Children.**

In a paper recently read before the British Medical Association, Dr. Francis Warner gave the results of his examination of 106 schools, containing in the aggregate 50,000 children. Among other conclusions arrived at by Dr. Warner was this, that when English and Irish children were compared with Jewish the latter were found superior, or in other words, freer from bodily defects and mental dulness, and endowed with greater powers of resistance. We are not disposed to question Dr. Warner's conclusions, but it would be satisfactory to have a little more detail. We would like, for example, also to know whether any comparison has been made between Jewish and Scottish children, or between the children of Northern England and those of Jews. It is to be remembered that the children of the second and third generation in the London slums are very sorry specimens of English boys and girls. The Jewish boys and girls of the same neighbourhoods have probably in most cases been quite recently imported from foreign countries, and consist of the first generation. All these, and other unstated facts, should be taken into consideration in making generalisations of the kind announced by Dr. Warner. It is a well-known fact that Jews marry "in and in" a very great deal; and one certainly does not expect their offspring, under such circumstances, to be bodily and mentally equal to English children, much less superior. The facts may be as Dr. Warner states, but at least we would like more information on the subject.

**The harmless, necessary Tart.**

Tomatoes are in season, and therewith has come again the cry that was raised last autumn, that the eating of them induces cancer. Cancer was as common in Britain long before tomatoes became cheap and popular, as it is now; but such a fact will make no impression on those who choose to see some connection between the two, and who will, perhaps, tell us next year that cabbages induce consumption and green peas lead to epilepsy. Without disputing the importance of diet both in health and disease, one may regret the numberless fads and caprices which in these days ban one food and "boom" another. That maltreatment of the digestive organs is at the root of many diseases is true enough; but it is to be remembered that to treat the average stomach as if it were an invalid is the best way to make it one. It is a popular notion that the stomachs of vegetarians undergo certain organic changes which make them more akin to the herbivorous animals. How far this idea is true no one who has not dissected a sworn vegetarian would dare to say; but there seems to be little doubt that a digestion which is never exercised on anything but the mildest meats becomes incapable of tackling anything stronger. Perhaps popular medical literature is partly to blame for the growing habit of over-nursing organs which are quite able to stand ordinary work. "Health articles" are written by doctors, and these, seeing people only when they are ill, forget that the papers they write for the "Family Journals" are read by men and women—especially women—who are perfectly well. "Avoid pastry" writes the doctor, thinking of the confirmed dyspeptic who left his consulting-room half an hour ago, and thereupon a hundred folks who were never a whit the worse for their tarts avoid pastry conscientiously and take to unending sago puddings, whose monotony their weary palate loathes. If we were to renounce all that we see or hear condemned as overstraining or misusing our digestive apparatus, we should probably take nothing but pepsin, with perhaps a little milk to exercise it on. There are times when after a too rigid dieting the most mature of us longs for the green apples and raspberry tarts of youth, and such a longing is an honest rebellion of the digestion against a regimen which keeps it weak for lack of proper exercise. To give a fair and reasonable consideration to the food we eat is a matter of common sense, but to make ourselves mentally the parallels of the monks of Mount Athos, and concentrate our attention on all that we should avoid, is to lay ourselves open to the chance of indigestion as much as if we indulged every day in the banquets of a Lucullus.

**An Imperial Medical "Home and Club."**

Dr. Withers Moore, speaking the other day as an official representative of the British Medical Association, expressed the hope that the sum of £60,000 possessed by the Association might be devoted to the purpose of erecting a suitable "Home" in the metropolis for all the members of the medical profession scattered throughout the Empire and the world. The idea has struck different members of the Association in different ways, as was to be expected. But there is a period of twelve years, during which the question may be fully considered on all sides, before any practical steps are imperatively required to be taken. The present writer is an appreciative member of the British Medical Association, and he feels somewhat warmly on the subject. Several alternative schemes have been put forward, and for some of them there is much to be said. But one cannot help feeling that such a cosmopolitan suggestion as that propounded by Dr. Withers Moore is of a nature to embrace and include all the other really worthy schemes hinted at by the various members of the Association. Let the members recall to mind that the British Medical Association has already established a Benevolent Fund, which does the best of work for aged and helpless medical men and their distressed families; that it has also established a Sickness and Annuity Fund, which is not only admirable, but unique; that it has provided its members with a journal which has no equal among professional journals for its price; and that it has a Parliamentary Bills Committee which guards the interests of the whole profession with rare intelligence and unceasing assiduity. All these things are and have been done mainly by the strong metropolitan centre of the Association; and without such a strong and intelligent centre they could not have been done at all. The "Home and Club" which it is proposed to provide is designed as a common meeting ground and protecting centre, not for the metropolis, but for every member of the British Medical Association, and, indeed, for every English speaking member of the medical profession throughout the world. At present there is no such central medical home anywhere. Is the Royal College of Physicians such a home? Or the Royal College of Surgeons? The suggestions provoke a smile! Medicine is now one of the most learned of all the professions. It is rapidly becoming more honoured than most. The remotest country practitioner has his share of the honour that falls to his calling, and should claim his rights as an integral part of the whole body medical. He owes it to himself to maintain a vital connection with the great and increasingly honourable profession of which he is a part. In no way can he so effectually accomplish this as by having a personal share and right in the one central home to which every member of the profession may properly claim to belong. It is a great, expansive, and imperial idea which Dr. Withers Moore has thrown out, and we cannot doubt that the imperial instincts of our great profession will soon be impatient to see it realised.

